# Intra-pancreatic fat deposition links to widespread systemic health risks: UK Biobank prospective cohort study

**DOI:** 10.1186/s13244-026-02206-7

**Published:** 2026-02-16

**Authors:** Yanna Cai, Nan Zhao, Jiarui Mi, Hanze Du, Ziqi Wan, Zhengye Liu, Yingyu Pan, Xiaxiao Yan, Zhengyang Fan, Jianing Li, Guanqiao Li, Venkata S. Akshintala, Xiaoyin Bai, Dong Wu

**Affiliations:** 1 Department of Gastroenterology, Peking Union Medical College Hospital, Chinese Academy of Medical Science and Peking Union Medical College, Beijing, China; 2 Department of Gastroenterology, The People’s Hospital of Tibetan Autonomous Region, Lhasa, China; 3 Institute of Clinical Medicine, National Infrastructures for Translational Medicine, Peking Union Medical College Hospital, Chinese Academy of Medical Science and Peking Union Medical College, Beijing, China; 4 Department of Gastroenterology, Sir Run Run Shaw Hospital, School of Medicine, Zhejiang University, Hangzhou, China; 5 Department of Endocrinology & Key Laboratory of Endocrinology of National Health Commission, Translation Medicine Centre, Peking Union Medical College Hospital, Beijing, China; 6 Department of Radiation Oncology, Cancer Hospital, Chinese Academy of Medical Sciences and Peking Union Medical College, Beijing, China; 7 Department of Plastic and Aesthetic Center, the First Affiliated Hospital, School of Medicine, Zhejiang University, Hangzhou, 310000 China; 8 Division of Gastroenterology, Department of Medicine, Johns Hopkins Medical Institution, Baltimore, MD USA; 9 Vanke School of Public Health and Institute for Healthy China, Tsinghua University, Beijing, Beijing, China

**Keywords:** Fatty pancreas, Systemic diseases, Mendelian randomization, Prospective cohort, Nonlinear association

## Abstract

**Introduction:**

Intra-pancreatic fat deposition (IPFD) is associated with pancreatic diseases, but its systemic implications remain unclear.

**Materials and methods:**

We analyzed 25,547 UK Biobank participants (median follow-up 6.27 years) with MRI-derived pancreatic proton density fat fraction. Multi-variable Cox models, causal mediation, restricted cubic splines, and subgroup analyses assessed IPFD–disease associations. Significant associations were examined through bidirectional Mendelian randomization (MR) using the UK Biobank and FinnGen data. Receiver operating characteristic curves and the Youden index were used to identify a clinically relevant and statistically optimal IPFD threshold.

**Results:**

Higher IPFD independently increased the risk of 12 multi-systemic diseases: non-insulin-dependent diabetes, primary hypertension, heart failure, cerebral infarction, cholelithiasis, gastritis and duodenitis, diaphragmatic hernia, chronic renal failure, gonarthrosis, disorders of refraction and accommodation, senile cataract, and sleep disorders. Causal mediation by non-insulin-dependent diabetes was negligible. Nonlinear dose–response patterns and effect modifications by sex, race, smoking, and obesity emerged. MR analysis supported the potential causal effects of IPFD on refractive/accommodation disorders and gonarthrosis. An IPFD cutoff of 7.35% (95% CI: 5.68–9.23%) optimally stratified the risk.

**Conclusion:**

IPFD is an independent risk factor for diverse conditions, including metabolic, cardiovascular, digestive, musculoskeletal, ophthalmologic, urinary, and mental/behavioral disorders. A pancreatic fat threshold of 7.35% may guide clinical screening and preventive strategies.

**Critical relevance statement:**

This study critically establishes intra-pancreatic fat as a novel, causal multi-system disease risk factor and provides a 7.35% quantitative threshold to advance radiological screening and prevention protocols.

**Key Points:**

- Limited research exists on the systemic effects of IPFD.
- Pancreatic fat deposition independently raises risk for 12 multi-system diseases.
- A 7.35% pancreatic fat threshold can guide clinical screening and prevention.

**Graphical Abstract:**

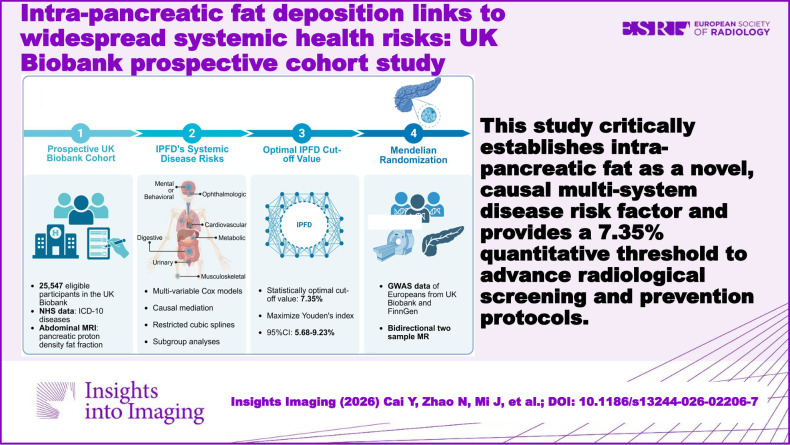

## Introduction

Intra-pancreatic fat deposition (IPFD) refers to the diffuse presence of fat in the pancreas [1, 2]. Excessive IPFD, which has a global prevalence of 16–33% [3, 4], is a concerning pathological condition. Several cohort studies and Mendelian randomization (MR) analyses have suggested that IPFD may play a causal role in the development of acute pancreatitis, chronic pancreatitis, and pancreatic ductal adenocarcinoma [5, 6]. The proton density fat fraction (PDFF), derived from magnetic resonance imaging (MRI), is a validated biomarker of IPFD, and accurately reflects the histological pancreatic fat fraction [7, 8].

The pancreas is an endocrine and exocrine gland crucial for substance and energy metabolism. Similar to hepatic fat deposition, IPFD, as a form of visceral and ectopic fat accumulation [9, 10], adversely affects the pancreas and may lead to extensive multi-system manifestations. A study has found a significant correlation between IPFD and circulating inflammatory biomarkers (soluble tumor necrosis factor receptors and CD163), indicating its role in chronic systemic inflammation [11]. While a handful of studies have explored the extra-pancreatic associations of IPFD, such as with COVID-19 [12, 13] and subclinical atherosclerosis [14], they are limited and primarily case-control studies. Furthermore, despite the increasing research focus on IPFD, a consensus regarding clinically meaningful thresholds for pancreatic fat fraction remains elusive, hampering the translation of research findings into clinical practice guidelines.

To bridge these gaps, we utilized the UK Biobank prospective cohort and pancreatic PDFF data to systematically investigate the associations between IPFD and systemic diseases.

## Material and methods

### Study design and population

This study utilized data from the UK Biobank, a large prospective cohort comprising over 500,000 participants aged 40–69 between 2006 and 2010, with linkage to National Health Service (NHS) data [15]. Participants who underwent abdominal MRI (April 2014–October 2024) and had quantifiable IPFD measurements were included. The initial MRI examination served as the baseline assessment. Follow-up time was calculated from the baseline MRI date until the first occurrence of any of the following: (1) first diagnosis of the disease of interest, (2) death from any cause, (3) loss to follow-up, or (4) the study end date (October 31, 2024). The extracted baseline characteristics included age, sex (assigned at birth), race, body mass index (BMI), obesity status (defined as BMI ≥ 30 kg/m²), smoking status, alcohol drinking status, physical activity (MET-min/week), and fatty liver presence (defined as liver PDFF ≥ 6.5%). Participants with missing IPFD values or incomplete covariate data were excluded. Regarding the definition of disease outcomes, please refer to Method S1.

This study was conducted under application number 100787 from the UK Biobank. Ethical approval and informed consent were provided by the UK Biobank study.

### MRI data acquisition and pancreatic fat quantification

All abdominal MRI data in this study were obtained from the UK Biobank [16, 17]. Pancreatic fat quantification data were obtained from the study by Yi Liu et al [18] Detailed methodological descriptions, including representative MRI images demonstrating the segmentation and quantification process, are available in the original publication and in Method S2.

### Statistical analysis methods

Comprehensive statistical methods were employed in this study, including descriptive analysis for baseline characteristics, Cox proportional hazards (PH) models for survival analysis, causal mediation analysis to explore mechanistic pathways [19], subgroup analyses stratified by demographic and clinical factors, restricted cubic splines (RCS) for nonlinear relationship assessment, MR analysis for causal inference [18, 20, 21], and ROC curve analysis for determining clinical cut-off values. All analyses were performed using R software (version 4.3.2) and the UK Biobank research analysis platform (RAP). A two-sided *p*-value < 0.05 was considered statistically significant. Detailed descriptions of all statistical methods are provided in Method S3.

## Results

### Characteristics of the study population in the UK Biobank

A total of 25,547 eligible UK Biobank participants were stratified into quartiles based on IPFD, revealing statistically significant differences in baseline characteristics, as summarized in Table 1. The median IPFD across the cohort was 8.02%. Those in the highest IPFD quartile (Q4) were notably older, predominantly male, and exhibited higher prevalence rates of obesity, previous or current smoking, and radiological fatty liver compared to the lowest quartile (Q1). Alcohol drinking history remained largely unchanged across quartiles, while median physical activity (metabolic equivalent (MET) min) declined from 2404 in Q1 to 1874 in Q4. Notably, despite this clear trend, two-thirds of the individuals in Q4 were not classified as obese, underscoring that elevated pancreatic fat does not uniformly equate to obesity.

**Table 1:** Baseline characteristics of eligible participants

Characteristic	Overall *N* = 25,547	Quartile of IPFD				*p*-value
		Q1 (lowest) *N* = 6387	Q2 *N* = 6387	Q3 *N* = 6386	Q4 (highest *N* = 6387	
Age (years), median (IQR)	64.00 (58.00–70.00)	61.00 (56.00–67.00)	63.00 (57.00–69.00)	65.00 (59.00–70.00)	67.00 (61.00–72.00)	< 0.001
Sex, *n* (%)						< 0.001
Female	12,971 (51)	4754 (74)	3490 (55)	2683 (42)	2044 (32)	
Male	12,576 (49)	1633 (26)	2897 (45)	3703 (58)	4343 (68)	
Race, *n* (%)						< 0.001
Non-white	1810 (7.1)	527 (8.3)	465 (7.3)	434 (6.8)	384 (6.0)	
White	23,737 (93)	5860 (92)	5922 (93)	5952 (93)	6003 (94)	
BMI (kg/m^2^), Median (IQR)	25.83 (23.47–28.76)	23.15 (21.51–25.17)	25.42 (23.47–27.77)	26.91 (24.68–29.58)	28.28 (25.80–31.15)	< 0.001
Obesity, *n* (%)	4550 (18)	215 (3.4)	755 (12)	1419 (22)	2161 (34)	< 0.001
Smoking status, *n* (%)						< 0.001
Never	16,080 (63)	4491 (70)	4112 (64)	3854 (60)	3623 (57)	
Previous or current	9467 (37)	1896 (30)	2275 (36)	2532 (40)	2764 (43)	
Alcohol drinking status, *n* (%)						0.23
Never	796 (3.1)	222 (3.5)	197 (3.1)	182 (2.8)	195 (3.1)	
Previous or current	24,751 (97)	6165 (97)	6190 (97)	6204 (97)	6192 (97)	
MET (min), median (IQR)	2146.00 (1093.00–3886.00)	2404.00 (1314.00–4239.00)	2213.00 (1116.00–3942.00)	2094.50 (1050.00–3786.00)	1874.00 (924.00–3546.00)	< 0.001
Fatty liver, *n* (%)	5094 (20)	271 (4.2)	987 (15)	1617 (25)	2219 (35)	< 0.001
IPFD, median (IQR)	8.02 (4.83–13.64)	3.52 (2.81–4.18)	6.31 (5.56–7.10)	10.30 (9.06–11.81)	19.94 (16.14–26.11)	< 0.001
Follow-up period, median (IQR), years	6.27 (5.65–7.10)	6.26 (5.67–7.08)	6.29 (5.67–7.13)	6.27 (5.64–7.08)	6.27 (5.62–7.08)	< 0.001

Continuous values were non-normally distributed and presented as median (interquartile range), and categorical variables were presented as counts (percentages)

*IPFD* intra-pancreatic fat deposition

### Elevated IPFD significantly increases the risk for 12 multi-system manifestations

**Fig. 1:**
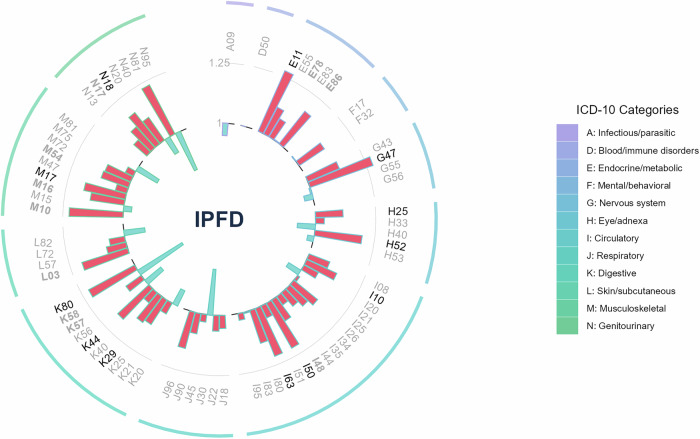
Circular barplot showing HRs for disease associations with IPFD. This circular barplot displays HRs representing the strength of associations between IPFD and various incident diseases across multiple systems. Each segment of the circular plot corresponds to a different disease, identified by International Classification of Diseases coding (ICD-10). The radial distance from the center represents the magnitude of the HR, with longer segments indicating stronger associations. Disease labels are color-coded to indicate statistical significance levels: black labels denote associations that remain significant after false discovery rate correction (FDR < 0.05), while bold gray labels indicate associations with unadjusted statistical significance (*p*-value < 0.05)

We systematically investigated the associations between IPFD and multi-system diseases using Cox PH models and extended Cox models with time-dependent covariates. To ensure sufficient statistical power while comprehensively exploring these relationships, we adjusted for a range of covariates, including age, sex, race, obesity, smoking status, alcohol drinking status, MET, and fatty liver. Adhering to the empirical rule of 10 events per candidate predictor parameter, 70 disease endpoints with ≥ 90 cases from the UK Biobank were analyzed. Twelve diseases significantly associated with IPFD were identified: non-insulin-dependent diabetes (NIDDM) (Hazard ratio [HR] 1.27, 95% CI: 1.16–1.40, FDR < 0.001), primary hypertension (HR 1.14, 95% CI: 1.07–1.21, FDR < 0.001), heart failure (HR 1.21, 95% CI: 1.08–1.36, FDR = 0.009), cerebral infarction (HR 1.21, 95% CI: 1.05–1.40, FDR = 0.041), cholelithiasis (HR 1.22, 95% CI: 1.08–1.37, FDR = 0.009), gastritis and duodenitis (HR 1.13, 95% CI: 1.05–1.23, FDR = 0.016), diaphragmatic hernia (HR 1.18, 95% CI: 1.08–1.28, FDR < 0.001), chronic renal failure (HR 1.17, 95% CI: 1.05–1.29, FDR = 0.021), gonarthrosis (HR 1.18, 95% CI: 1.07–1.29, FDR = 0.009), disorders of refraction and accommodation (HR 1.20, 95% CI: 1.10–1.30, FDR < 0.001), senile cataract (HR 1.11, 95% CI: 1.05–1.19, FDR = 0.009), and sleep disorders (HR 1.28, 95% CI: 1.08–1.51, FDR = 0.025). The detailed results are visualized in Fig. 1 and provided in Table S1. These findings indicate that a higher IPFD is significantly associated with an increased risk of multiple metabolic, cardiovascular, respiratory, digestive, musculoskeletal, and neurological disorders.

To validate the robustness of these results, we performed a sensitivity analysis, restricting the definition of a positive outcome to the first disease occurrence recorded ≥ 12 weeks after baseline. Consistent results were observed in the sensitivity analysis (Table S2).

### IPFD is directly linked to multi-system manifestations, bypassing NIDDM

**Fig. 2:**
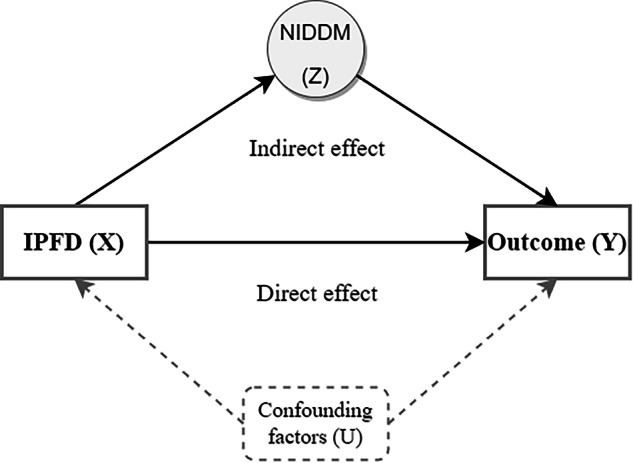
Conceptual framework for causal mediation analysis. This diagram illustrates the theoretical model used to examine causal pathways between IPFD and health outcomes. The framework shows IPFD as the exposure variable (X) and various disease outcomes as dependent variables (Y). The model decomposes the relationship into two distinct pathways: a direct effect from IPFD to outcomes, and an indirect effect mediated through Non-Insulin Dependent Diabetes Mellitus (NIDDM) as an intermediate variable (Z). Arrows indicate the direction of proposed causal relationships. Confounding variables (U) that may influence both exposure-mediator and exposure-outcome relationships are represented by dashed lines

Given the established association between IPFD and NIDDM, along with the systemic disease risks linked to NIDDM, we employed causal mediation analysis to investigate whether the effect of IPFD on multi-system manifestation risk operates through NIDDM as a mediating pathway. As illustrated in Fig. 2, for each outcome, we constructed a cohort free of NIDDM and the outcome disease at baseline, defined NIDDM occurring before the outcome disease as the mediating variable, and applied logistic regression models adjusted for confounding factors. The causal steps approach was used to test the mediation effects.

Our analysis revealed no statistically significant indirect effect of IPFD on systemic manifestation risk mediated through NIDDM (Table 2). Specifically, across all 11 diseases examined, the *p*-values for ACEM were all greater than 0.05. The proportion of effects mediated by NIDDM (Prop. Mediated) was consistently low, with most diseases showing mediation proportions < 2%. This indicates that NIDDM does not function as a causal mediator in the association between IPFD and multi-system manifestations. The direct effect of IPFD on disease risk remained robust, with ADE being statistically significant across all diseases (*p* < 0.05), suggesting that IPFD influences outcomes predominantly via pathways independent of NIDDM.

**Table 2:** Causal mediation analyses of NIDDM in IPFD-manifestation associations across systems

Category	Disease	ICD-10	ACME	*p* (ACME)	ADE	*p* (ADE)	Prop. Mediated (%)	*p* (Prop.)	Total effect
Cardiovascular	Heart failure	I50	1.0000 (1.0000 to 1.0001)	0.114	1.0016 (1.0007 to 1.0026)	< 0.001	1.67 (−0.28 to 8.45)	0.114	1.0017 (1.0007 to 1.0027)
	Primary hypertension	I10	1.0000 (1.0000 to 1.0002)	0.43	1.0065 (1.0031 to 1.0101)	< 0.001	0.45 (−0.56 to 3.46)	0.43	1.0065 (1.0031 to 1.0101)
Cerebrovascular	Cerebral infarction	I63	1.0000 (1.0000 to 1.0001)	0.894	1.0013 (1.0003 to 1.0023)	0.004	−0.10 (−2.64 to 5.35)	0.894	1.0013 (1.0003 to 1.0023)
Gastrointestinal	Cholelithiasis	K80	1.0000 (1.0000 to 1.0001)	0.13	1.0023 (1.0009 to 1.0037)	< 0.001	1.02 (−0.14 to 5.96)	0.13	1.0023 (1.0010 to 1.0038)
	Diaphragmatic hernia	K44	1.0000 (1.0000 to 1.0002)	0.16	1.0040 (1.0017 to 1.0063)	< 0.001	0.90 (−0.23 to 4.69)	0.16	1.0040 (1.0018 to 1.0064)
	Gastritis and duodenitis	K29	1.0000 (1.0000 to 1.0001)	0.226	1.0033 (1.0011 to 1.0058)	0.002	0.93 (−0.68 to 5.39)	0.228	1.0033 (1.0011 to 1.0058)
Musculoskeletal	Gonarthrosis	M17	1.0000 (0.9999 to 1.0001)	0.62	1.0023 (1.0007 to 1.0041)	0.01	−0.43 (−4.07 to 2.86)	0.632	1.0023 (1.0007 to 1.0041)
Neurological	Sleep disorders	G47	1.0000 (1.0000 to 1.0001)	0.948	1.0011 (1.0004 to 1.0020)	0.008	−0.04 (−2.20 to 5.26)	0.946	1.0011 (1.0004 to 1.0020)
Ophthalmic	Disorders of refraction and accommodation	H52	1.0000 (1.0000 to 1.0001)	0.906	1.0035 (1.0016 to 1.0056)	< 0.001	0.04 (−1.39 to 2.64)	0.906	1.0035 (1.0016 to 1.0056)
	Senile cataract	H25	1.0000 (0.9999 to 1.0001)	0.944	1.0037 (1.0013 to 1.0061)	0.002	0.03 (−1.91 to 3.04)	0.946	1.0037 (1.0013 to 1.0061)
Renal	Chronic renal failure	N18	1.0000 (1.0000 to 1.0001)	0.462	1.0015 (1.0003 to 1.0028)	0.012	0.79 (−1.60 to 8.56)	0.466	1.0015 (1.0003 to 1.0029)

All models adjusted for age, sex, race, obesity status, smoking status, alcohol consumption status, METs, and fatty liver

*ACME* average causal mediation effect (indirect effect through NIDDM), expressed as odds ratio (OR), *ADE* average direct effect (effect of IPFD not mediated by NIDDM), expressed as OR, *IPFD* intra-pancreatic fat deposition, *Prop. Mediated* proportion of total effect mediated by NIDDM, *NIDDM* non-insulin-dependent diabetes mellitus

### Subgroup analyses reveal effect modifications by sex, race, smoking, and obesity

To assess potential effect modification by key demographic and lifestyle factors, we conducted subgroup analyses of the 12 IPFD-associated manifestations. While the associations between IPFD and most diseases remained relatively consistent across subgroups, several significant interactions emerged (see Table S3). For NIDDM, the association was stronger in females (HR 1.49, 95% CI: 1.25–1.78) than in males (HR 1.20, 95% CI: 1.07–1.35; *p*-interaction = 0.006). For sleep disorders, the risk in white individuals was higher (non-white: HR 0.62, 95% CI: 0.16–2.30; white: HR 1.30, 95% CI: 1.10–1.54; *p*-interaction = 0.037). Smoking status modification was observed for gastritis and duodenitis, with stronger associations among never smokers (never smoker: HR 1.20, 95% CI: 1.08–1.32 vs ever smoker: HR 1.05, 95% CI: 0.92–1.19, *p*-interaction = 0.038). Obesity modified associations for cholelithiasis (BMI < 30: HR 1.28, 95% CI: 1.11–1.48; BMI ≥ 30: HR 1.09, 95% CI: 0.90–1.33; *p*-interaction = 0.023) and gonarthrosis (BMI < 30: HR 1.26, 95% CI: 1.12–1.41; BMI ≥ 30: HR 1.04, 95% CI: 0.89–1.22; *p*-interaction = 0.010), with IPFD associations being more pronounced among non-obese individuals.

### Unveiling nonlinear link patterns between IPFD and diseases via RCS

**Fig. 3:**
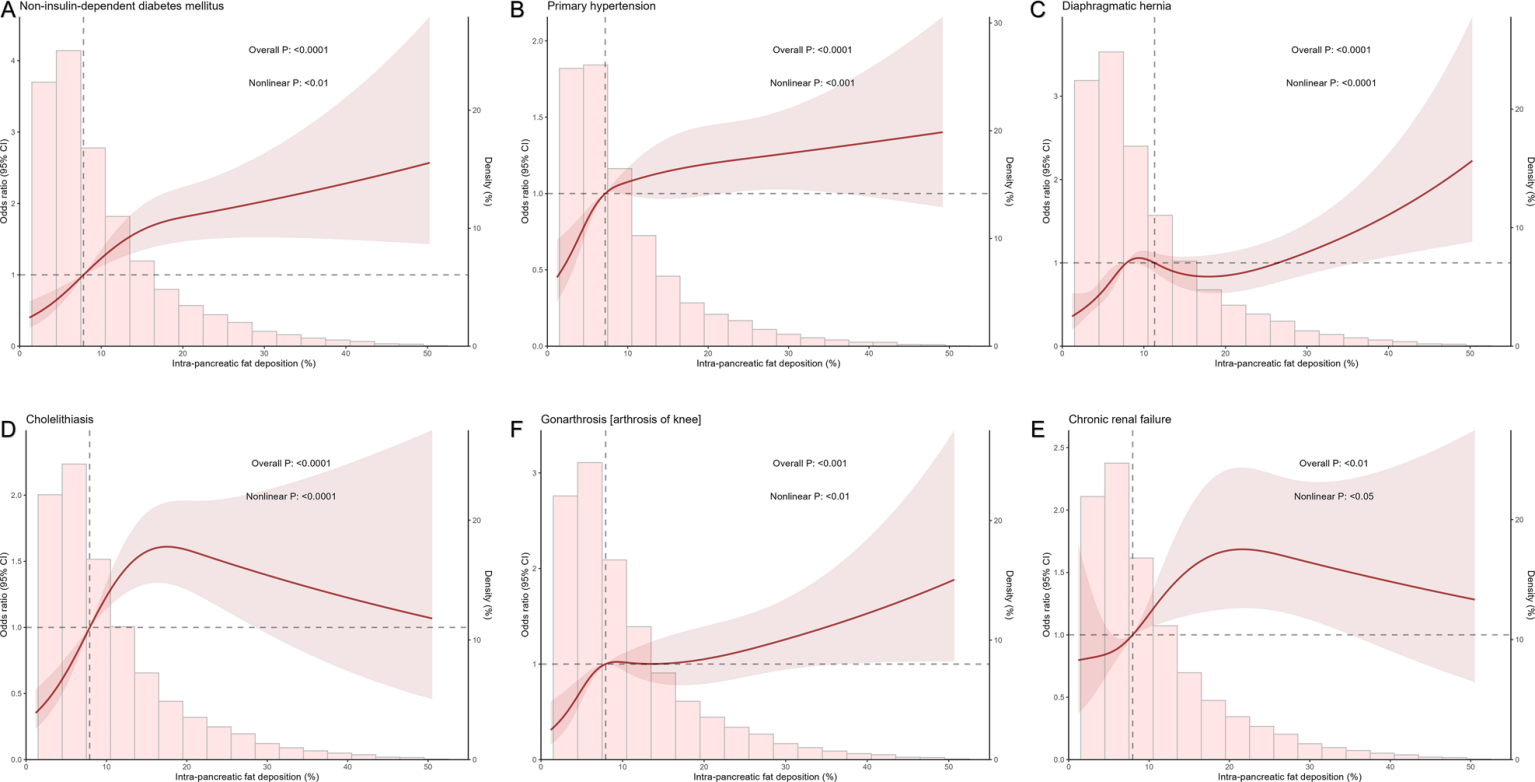
Nonlinear associations between IPFD and disease risks. **A**–**F** This figure presents restricted cubic spline analyses demonstrating nonlinear associations between IPFD and disease risk for six conditions. Each panel shows a smooth curve representing relative ORs with 95% confidence intervals (CIs) plotted against IPFD percentage values. Histograms in the background of each panel show the distribution of IPFD values in the study population. Shaded areas around the curves represent CIs. Statistical significance indicators (overall *p*-values and nonlinear *p*-values) are displayed for each condition

We further investigated the potential nonlinear relationships between IPFD and 12 diseases using RCS, which can detect complex patterns often missed by traditional linear models by fitting piecewise cubic polynomials between predefined knots [22]. Our analysis screened out six diseases that may have a nonlinear association (nonlinear *p*-value < 0.05), and three distinct nonlinear patterns emerged (Figs. 3 and S1).

For NIDDM and primary hypertension, the odds ratio (OR) exhibited a continuous upward trend with increasing IPFD, with peak slopes at IPFD levels of 7.48% for NIDDM and 4.13% for primary hypertension. Subsequently, the rate of increase gradually diminished as the IPFD rose, stabilizing at IPFD levels of 21.29% and 25.30%, respectively, as demonstrated by the RCS curves transitioning from a steep rise to a plateau. For diaphragmatic hernia and gonarthrosis, the OR followed a complex, non-monotonic pattern: an initial increase, a slight decline, and a subsequent rise. Specifically, diaphragmatic hernia showed a peak slope at an IPFD of 6.21%, reaching a nadir at 11.71% before rising again, while gonarthrosis exhibited a peak slope at an IPFD of 4.66%, dipping at 10.46% before subsequent elevation. For cholelithiasis and chronic renal failure, the OR initially increased and then decreased with rising IPFD. Cholelithiasis demonstrated a peak slope at an IPFD of 7.37% with a maximum OR occurring at 17.49%, while chronic renal failure showed a peak slope at an IPFD of 10.08% with a maximum OR at 21.58%.

### Bidirectional MR supports causal effects of IPFD on ocular and joint disorders

**Fig. 4:**
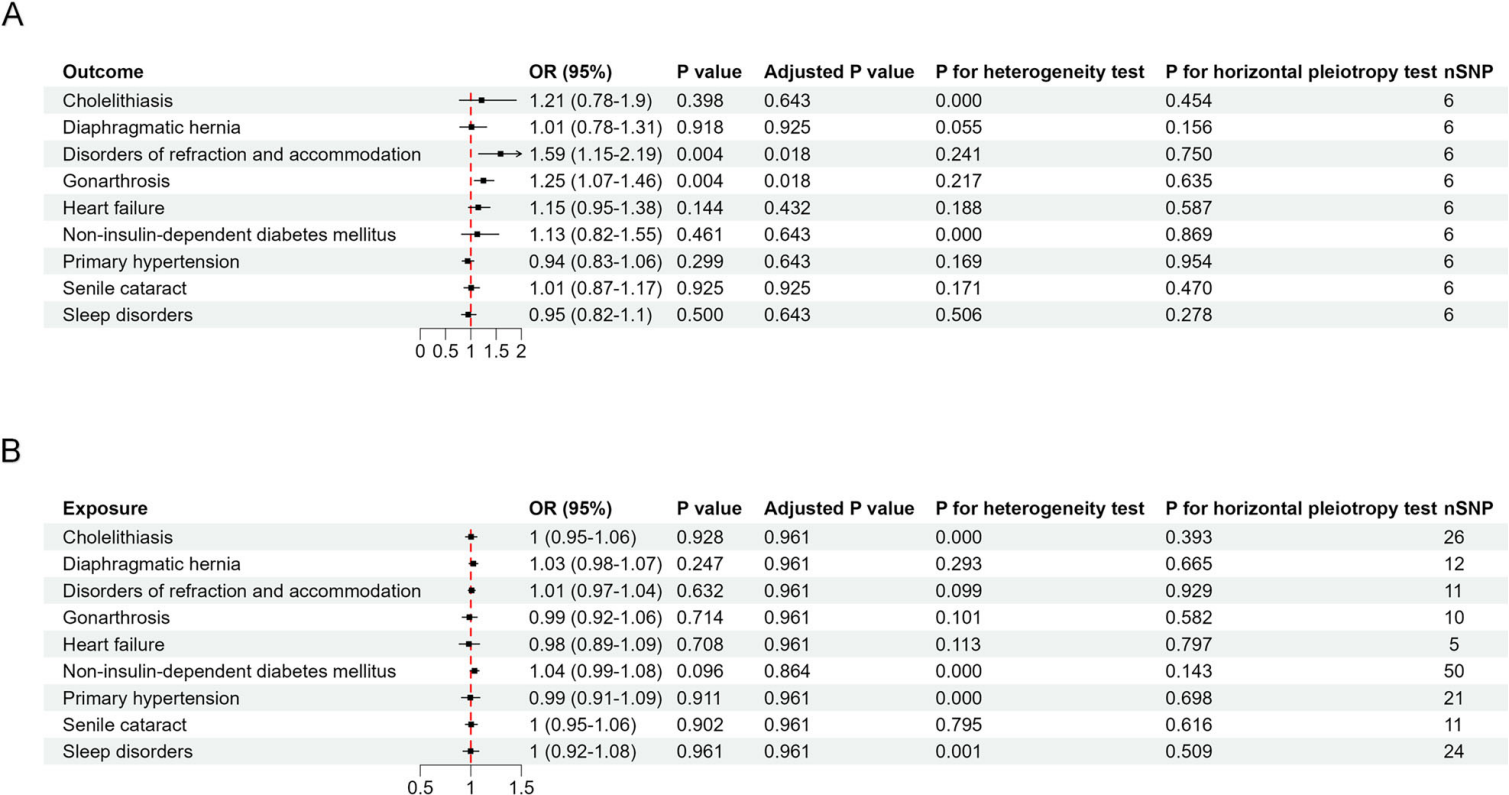
Bidirectional MR analysis results. This figure presents results from bidirectional MR analyses examining causal relationships between IPFD and associated diseases. **A** Shows analyses with IPFD as the exposure variable. **B** Displays reverse analyses with diseases as exposures and IPFD as the outcome variable. Results are presented as forest plots showing ORs with 95% CIs for each disease-IPFD relationship. The plots include both unadjusted and multiple testing-corrected *p*-values

**Fig. 5:**
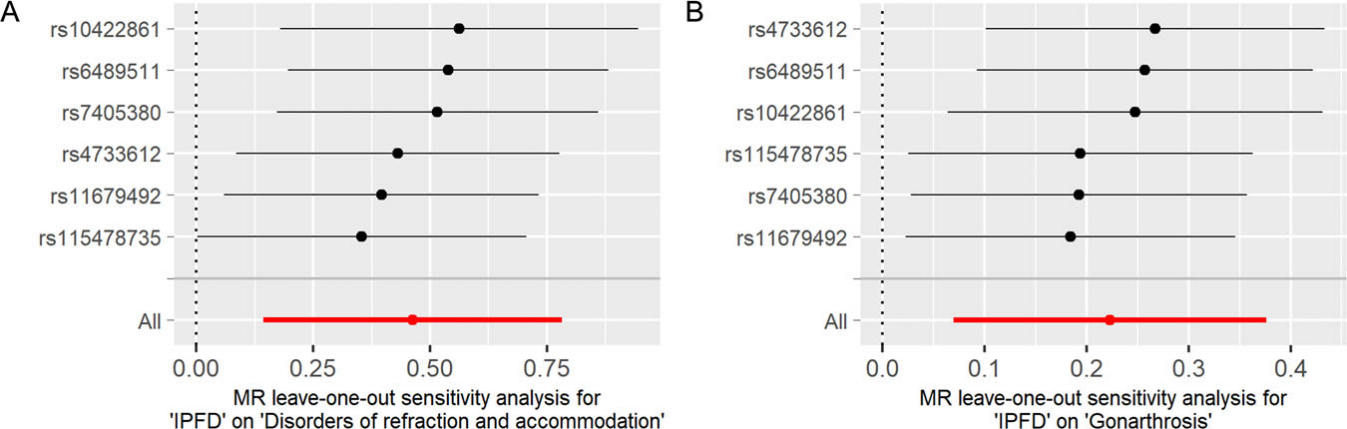
Leave-one-out sensitivity analysis for MR results. The figure presents the sensitivity analysis results of causal relationships between IPFD and two diseases: disorders of refraction and accommodation (**A**), and gonarthrosis (**B**). Each row shows the MR estimate (effect size) and 95% CI when excluding one specific SNP from the analysis, with individual SNPs identified by their rsID numbers. The bottom row represents the overall MR estimate using all six IVs SNPs

We further conducted a bidirectional two-sample MR analysis to assess the associations between IPFD and related diseases. We obtained genome-wide association study (GWAS) summary data for 9 out of 12 diseases from the FinnGen database (Table S4). Data on cerebral infarction, gastritis and duodenitis, and chronic renal failure were unavailable. After screening, we retained 6 single-nucleotide polymorphisms (SNPs) as instrumental variables (IVs) for IPFD (Table S5). MR results (Fig. 4) revealed significant causal effects of IPFD as exposure on disorders of refraction and accommodation (OR = 1.59, 95% CI: 1.15–2.19, adjusted *p*-value = 0.018), and gonarthrosis (OR = 1.25, 95% CI: 1.07–1.46, adjusted *p*-value = 0.018). No significant horizontal pleiotropy was detected. Leave-one-out sensitivity analyses (Fig. 5) confirmed the robustness of these findings, with all SNPs exhibiting effect estimates that were directionally consistent with the primary analysis. Reverse MR analyses showed no causal effects of other diseases on IPFD.

### Determination of a clinically meaningful IPFD cut-off value

First, we constructed a new composite outcome indicator based on the association between IPFD and 12 diseases: the presence of any IPFD-related disease. We used ROC curves to evaluate the diagnostic performance of IPFD for the composite outcome and identified the IPFD value that maximizes Youden’s index [23]. The optimal cut-off value determined by Youden’s index method was 7.35% (95% CI: 5.68–9.23%) (Fig. S2). Simple logistic regression models using only the binary IPFD cut-off yielded area under the receiver operating characteristic curve (AUCs) ranging from 0.54 for diaphragmatic hernia to 0.71 for non-insulin-dependent diabetes mellitus (NIDDM), all exceeding the random-chance threshold of 0.5, indicating its potential clinical utility (Table S6). Additionally, the disease-specific optimal IPFD cut-off values for the 12 individual outcomes ranged from 6.20% for diaphragmatic hernia to 12.14% for heart failure (Fig. S3).

### Discussion

This large-scale prospective cohort study provides comprehensive evidence of the systemic implications of IPFD. Our findings revealed that elevated IPFD was independently associated with an increased risk of 12 multi-system diseases: NIDDM, primary hypertension, heart failure, cerebral infarction, cholelithiasis, gastritis and duodenitis, diaphragmatic hernia, chronic renal failure, gonarthrosis, disorders of refraction and accommodation, senile cataract, and sleep disorders. The potential causal effects of IPFD on refractive and accommodation disorders, and gonarthrosis were further supported by MR analysis. These conditions spanned metabolic, cardiovascular, digestive, musculoskeletal, ophthalmologic, urinary, and mental/behavioral disorders. Notably, these associations persisted after adjusting for traditional risk factors, including obesity, smoking, alcohol consumption, and fatty liver disease, suggesting that excessive pancreatic fat represents an independent pathophysiological entity with broad systemic implications.

Among the identified associations, the relationship between IPFD and NIDDM warrants particular attention and has been extensively documented in the literature [24–26]. NIDDM is widely acknowledged as having heterogeneous roots [27], although epidemiological studies consistently show a strong correlation with overnutrition [28, 29]. Roy Taylor’s “twin cycle hypothesis” posits that self-reinforcing fat accumulation cycles within the liver and pancreas, driven by a chronic positive calorie balance, may explain NIDDM development [30, 31]. Energy restriction can normalize β-cell function by reducing IPFD [32]. The hypothesis also points out that the role of excess fat inside the liver and pancreas in the genesis of NIDDM is contingent on exceeding a personal fat threshold [30, 33]. Interestingly, our study revealed that females were at a higher risk of developing NIDDM with increased IPFD. This observed sex disparity warrants careful consideration. Men and women exhibit distinct patterns of fat distribution: males tend toward visceral fat accumulation, while females predominantly display subcutaneous fat distribution. Females may possess a lower pancreatic fat threshold, meaning that a relatively smaller absolute increase in IPFD could be sufficient to trigger β-cell dysfunction and hyperglycemia. Our findings aligned with the “personal fat threshold” theory and further suggested that sex may be a modulating factor for this threshold. In addition to sex differences, recent evidence highlights racial disparities in pancreatic fat deposition. A 2025 UK Biobank analysis found that White individuals had the highest pancreatic fat fraction among nondiabetic participants, a trend also observed in those with diabetes [34]. These findings suggest that racial background may influence pancreatic fat content and modulate the risk of IPFD-related diseases.

The impacts and mechanisms of IPFD on the other identified systemic diseases remained relatively underexplored. Through causal mediation analysis, we confirmed that these associations were independent of NIDDM effects, indicating that IPFD may possess independent pathophysiological effects beyond diabetes pathogenesis. Notably, the association between IPFD and the risk of cardiovascular diseases (CVDs), such as primary hypertension, heart failure, and cerebral infarction, merits special emphasis. A meta-analysis published in 2025 involving over 7000 participants demonstrated that a high IPFD was significantly correlated with increased aortic intima-media thickness (IMT), elevated carotid IMT, and enhanced vascular stiffness [35]. This finding is consistent with our results, highlighting the necessity of investigating IPFD as an additional risk factor for CVDs, independent of general obesity and type 2 diabetes. A possible explanation for these aforementioned associations is aggravated chronic systemic inflammation due to abnormal fat accumulation in the pancreas. Elevated levels of circulatory pro-inflammatory cytokines such as leptin and tumor necrosis factor alpha (TNF-α) in populations with fatty pancreas may suggest a link between IPFD and systemic inflammation [11, 36].

Subgroup analysis and RCS analyses revealed complex disease-specific association patterns. The RCS model identified three distinct non-linear association patterns among the six diseases, and differences in the modifying effects of demographic and lifestyle factors across different diseases were observed. The high complexity of the associations between IPFD and diseases was manifested not only in the significant risk differences among different population subgroups but also in the sophisticated non-monotonic dose-response relationships. These findings indicated the remarkable heterogeneity in the impacts and mechanisms of IPFD on different diseases. Further in-depth studies are required in the future to clarify the specific mechanisms underlying these associations.

This study represents the largest and most comprehensive investigation to date on the systemic implications of IPFD, spanning a wide spectrum of disease categories. Its strengths include extensive subgroup analyses, exploration of non-linear relationships, and the incorporation of MR to strengthen causal inference. Furthermore, by constructing a composite outcome indicator based on 12 IPFD-related diseases, this study established a statistically optimal IPFD cut-off value of 7.35% (95% CI: 5.68–9.23%), which offers a clinically actionable threshold for pancreatic fat fraction as a biomarker and addresses a critical gap in the field.

Our study has several limitations. First, reliance on European-population databases, such as the UK Biobank and FinnGen, may limit generalizability to other ethnicities. Second, MR analyses are subject to inherent limitations, including the assumption of direct causality, which may not hold when pathophysiological mechanisms are complex or unknown. The identified genetic associations may operate through indirect pathways or reflect pleiotropic effects, and our analyses may not capture the non-linear causal relationships suggested by the cohort analyses. Therefore, while our MR results support potential causal associations, they should be interpreted cautiously and require validation through mechanistic studies. Third, several diseases examined in this study, including diabetes, hypertension, cholelithiasis, gastritis, and gonarthrosis, are often underdiagnosed in clinical practice. This introduces the possibility of reverse causation bias, wherein prevalent but undiagnosed disease at the time of MRI assessment may have influenced IPFD levels. To mitigate this concern, we performed sensitivity analyses excluding disease events occurring within 12 weeks of baseline, which yielded consistent results. Nevertheless, this approach cannot fully exclude the possibility of an undiagnosed prevalent disease. Future studies incorporating more comprehensive baseline screening would better distinguish incident from prevalent undiagnosed cases. Additionally, surveillance bias should be considered, as individuals with elevated IPFD or related metabolic abnormalities may undergo more frequent medical assessments, potentially increasing disease detection. Fourth, the derived 7.35% IPFD threshold needs external validation across diverse populations owing to potential heterogeneity.

In conclusion, our study is the first to use a prospective cohort and MR to provide a comprehensive overview of the multi-system manifestation risks associated with IPFD. By clarifying these associations, our findings lay a solid foundation for incorporating pancreatic fat quantification into risk stratification and for guiding future research on pancreatic fat metabolism and its systemic health impacts.

## Supplementary information

Supplementary information

Supplementary information

## Data Availability

This study used publicly available databases and resources: GWAS data from FinnGen, https://www.finngen.fi/en; LDlink tools, https://ldlink.nih.gov; and R Project, https://www.r-project.org. The cohort data that support the findings of this study are available from the UK Biobank. Restrictions apply to the availability of these data, which were used under license for this study. Data are available from https://www.ukbiobank.ac.uk/ with the permission of the UK Biobank. The analytical code (R scripts) used in this study is publicly available at: https://github.com/TGOOAA2/IPFD/. Most analyses require execution on the UK Biobank RAP due to data access restrictions and computational dependencies.

## Abbreviations

ACME: Average causal mediation effect
ADE: Average direct effect
AUC: Area under the ROC curve
BMI: Body mass index
CI: Confidence interval
CVD: Cardiovascular diseases
FDR: False discovery rate
GWAS: Genome-wide association study
HR: Hazard ratio
ICD-10: International classification of diseases 10th revision
IMT: Intima-media thickness
IPFD: Intra-pancreatic fat deposition
IV: Instrumental variable
MET: Metabolic equivalent
MR: Mendelian randomization
MRI: Magnetic resonance imaging
NHS: National Health Service
NIDDM: Non-insulin-dependent diabetes mellitus
OR: Odds ratio
PDFF: Proton density fat fraction
PH: Proportional Hazards
RAP: Research analysis platform
RCS: Restricted cubic splines
ROC: Receiver operating characteristic
SNP: Single-nucleotide polymorphism
TNF-α: Tumor necrosis factor alpha
