# Supplementary material for: Intra-pancreatic fat deposition links to widespread systemic health risks: UK Biobank prospective cohort study
Source: Insights Imaging. 2026 Feb 16;17:48. doi: 10.1186/s13244-026-02206-7 (PMC12909717; doi:10.1186/s13244-026-02206-7)
Supplement: Supplementary file 1 — Supplementary information [file 13244_2026_2206_MOESM1_ESM.pdf]

Intra-pancreatic fat deposition links to widespread systemic health risks: UK Biobank prospective cohort study

ELECTRONIC SUPPLEMENTARY MATERIAL

Method S1. Disease Outcome Definition

Disease outcomes were identified through the UK Biobank's electronic health record system throughout the follow-up period, including hospital admission records, death registrations, primary care data and and patient-reported outcomes. Disease diagnoses were standardized based on International Classification of Diseases coding (ICD-10) codes. Disease outcome data in the UK Biobank are continuously updated through regular linkage to NHS records, ensuring new diagnoses are captured throughout the follow-up period.

Method S2. Pancreatic Fat Quantification Workflow

All abdominal MRI data in this study were obtained from the UK Biobank. All abdominal scans were performed using a Siemens Aera 1.5T scanner (Syngo MRI D13; Siemens, Erlangen, Germany).[1] For volumetric evaluation of the pancreas, a three-dimensional volumetric interpolated breath-hold examination sequence was acquired in transverse orientation.[2] A single breath-hold cardiac-

gated shortened Modified Look-Locker Inversion Recovery sequence was acquired for T1 mapping (spatial resolution = 1.146×1.146×8 mm^3, matrix = 384×288×7, TE/TR = 1.93/480.6 ms, flip angle α = 35°, acceleration factor R = 2).[2] A multi-echo sequence with 10 different echoes was employed to enable measurements of iron and fat content.[2] Pancreatic fat quantification data were obtained from the study by Yi Liu et al, which employed a validated deep learning pipeline for automated pancreatic segmentation and fat quantification from UK Biobank MRI data.[3] The specific process is as follows. First, on high-resolution T1-weighted three-dimensional volumetric interpolated breath-hold examination (3D VIBE) sequence images, a deep learning model based on a modified U-Net architecture was employed for automated three-dimensional whole-organ pancreas segmentation. This model had been trained on a large volume of manually annotated data. Subsequently, this 3D segmentation mask was resampled and precisely mapped onto single-slice multi-echo Dixon proton density fat fraction (PDFF) images, thereby defining the pancreatic region of interest (ROI) at the corresponding level. The entire image processing and analysis pipeline was subject to rigorous quality control. All automated segmentation results underwent multi-stage manual visual review and confirmation by experienced analysts. Image analysis was performed by a team of radiographers trained according to unified standard operating procedures.

Method S3. Statistical Analysis Methods

Descriptive Analysis

Non-normally distributed continuous variables were presented as median (interquartile range), normally distributed continuous variables as mean (standard deviation), and categorical variables as frequency (percentage). The normality of continuous variables was assessed using the Kolmogorov-Smirnov test. Inter-group comparisons employed the Kruskal-Wallis test for non-normally distributed continuous variables, ANOVA for normally distributed continuous variables, and Pearson's chi-square test for categorical variables.

Survival Analysis

Cox proportional hazards models were employed to assess associations between IPFD and disease incidence. The proportional hazards (PH) assumption was verified using Schoenfeld residuals. When the PH assumption was violated, extended Cox models incorporating time-dependent covariates were implemented. The Benjamini-Hochberg method was used to control the false discovery rate (FDR). Sensitivity analyses were performed to ensure the robustness.

Causal Mediation Analysis

Logistic regression models adjusting for confounding factors were fitted for the mediating variable and the outcome variable, respectively. Then, potential outcomes were generated through a quasi-Bayesian Monte Carlo simulation. The average causal mediation effect (ACME), the average direct effect (ADE),

the total effect was decomposed (total effect = ACME + ADE), and the mediation effect (ACME/Total Effect) were calculated[4].

Subgroup Analysis

Subgroup analyses were grouped by sex, age (≥60 years and <60 years), race, smoking status, alcohol consumption status, and obesity status. The p-value for interaction was calculated using the likelihood ratio test.

Nonlinear Relationship Analysis

The restricted cubic splines (RCS) method was employed to explore potential nonlinear relationships between the IPFD and disease risk.

Mendelian Randomization Analysis

Genome-wide association study (GWAS) summary statistics for IPFD were obtained from a prior UK Biobank study[4], while GWAS data for IPFD related diseases were sourced from the FinnGen database[5]. All GWAS datasets originated from European-ancestry populations with minimal sample overlap. Instrumental variables (IVs) were selected using a genome-wide significance threshold ($p < 5e-8$). When fewer than four IVs were identified, this threshold was systematically relaxed to a p-value < 5e-7 or 5e-6. Palindromic single-nucleotide polymorphisms (SNPs) were excluded. Potential pleiotropic effects were assessed using LDtrait and GWAS catalog, by cross-referencing all instrumental SNPs against both classical confounders (e.g., smoking, alcohol consumption, BMI), and against a wide range of disease outcomes in the GWAS catalog. No SNPs were found to have robust primary associations with

the specific diseases under investigation[6]. The inverse-variance weighted (IVW) method served as the primary analytical approach. Random-effects models were employed when significant heterogeneity was detected; otherwise, fixed-effects models were applied. Horizontal pleiotropy was assessed via MR-Egger intercept testing. Multiple testing correction was performed using the Benjamini-Hochberg method, and leave-one-out sensitivity analyses were conducted.

Determination of Clinical Cut-off

Logistic regression models were fitted to assess the relationship between IPFD and the composite outcome (the presence of any IPFD-related disease). Receiver operating characteristic (ROC) curves were generated, and the optimal IPFD cut-off value maximizing the Youden index was identified. Confidence intervals were estimated using the bootstrap resampling method with bias-corrected and accelerated (BCa) adjustment. For each disease and the composite outcome, the data were randomly split into a training set (70%) and a test set (30%). The predictive performance of the optimal IPFD cut-off was evaluated using a simple logistic regression model with IPFD status (above vs. below cut-off) as the only predictor variable, assessed on an independent test set after model training on the training set. Additionally, to provide disease-specific clinical guidance, we calculated optimal IPFD cut-off values for each of the 12 individual diseases using the same method.

Statistical Software

Statistical analyses were conducted using R software (version 4.3.2) and the UK Biobank Research Analysis Platform (RAP). A two-sided p-value < 0.05 was considered statistically significant.

Reference

1. P T, C M, Og M, et al. Association of Pancreas Volume With Adverse Clinical Outcomes. *J Am Heart Assoc*. 2025;14(21). doi:10.1161/JAHA.125.042348

2. Kongara T, Carruth A, Bhat P, Virostko J. Determinants of Pancreatic Size and Fat Content in the UK Biobank: Influence of Race, Genetic Variants, and Risk Factors. *J Clin Endocrinol Metab*. 2025;110(12):e3945-e3951. doi:10.1210/clinem/dgaf420

3. Liu Y, Basty N, Whitcher B, et al. Genetic architecture of 11 organ traits derived from abdominal MRI using deep learning. *eLife*. 2021;10:e65554. doi:10.7554/eLife.65554

4. Tingley D, Yamamoto T, Hirose K, Keele L, Imai K. **mediation** : *R* Package for Causal Mediation Analysis. *J Stat Softw*. 2014;59(5). doi:10.18637/jss.v059.i05

5. Kurki MI, Karjalainen J, Palta P, et al. FinnGen provides genetic insights from a well-phenotyped isolated population. *Nature*. 2023;613(7944):508-518. doi:10.1038/s41586-022-05473-8

6. Burgess S, Cronjé HT. Incorporating biological and clinical insights into variant choice for Mendelian randomisation: examples and principles. *eGastroenterology*. 2024;2(1). doi:10.1136/egastro-2023-100042

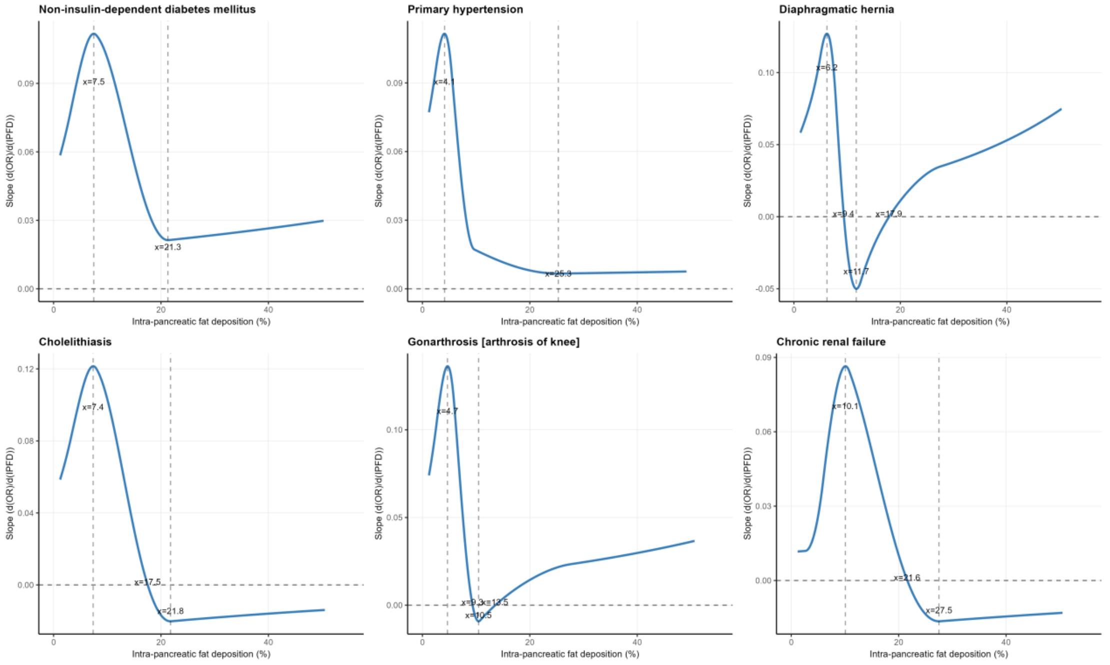

Figure S1. Slope plots of restricted cubic spline (RCS) analysis for nonlinear associations between intra-pancreatic fat deposition and disease risk. This figure presents slope plots derived from RCS analysis, complementing the nonlinear associations shown in Figure 3. These plots illustrate the slope of the derivative of the odds ratio (d(OR)/d(IPFD)) with respect to IPFD, expressed as a percentage, for six diseases with significant nonlinear relationships. The x - axis represents IPFD percentage, and the y - axis shows the slope of the OR change per unit IPFD. Vertical dashed lines and annotated “x” values indicate critical knots in the RCS model, marking transitions in the direction or magnitude of the association between IPFD and disease risk.

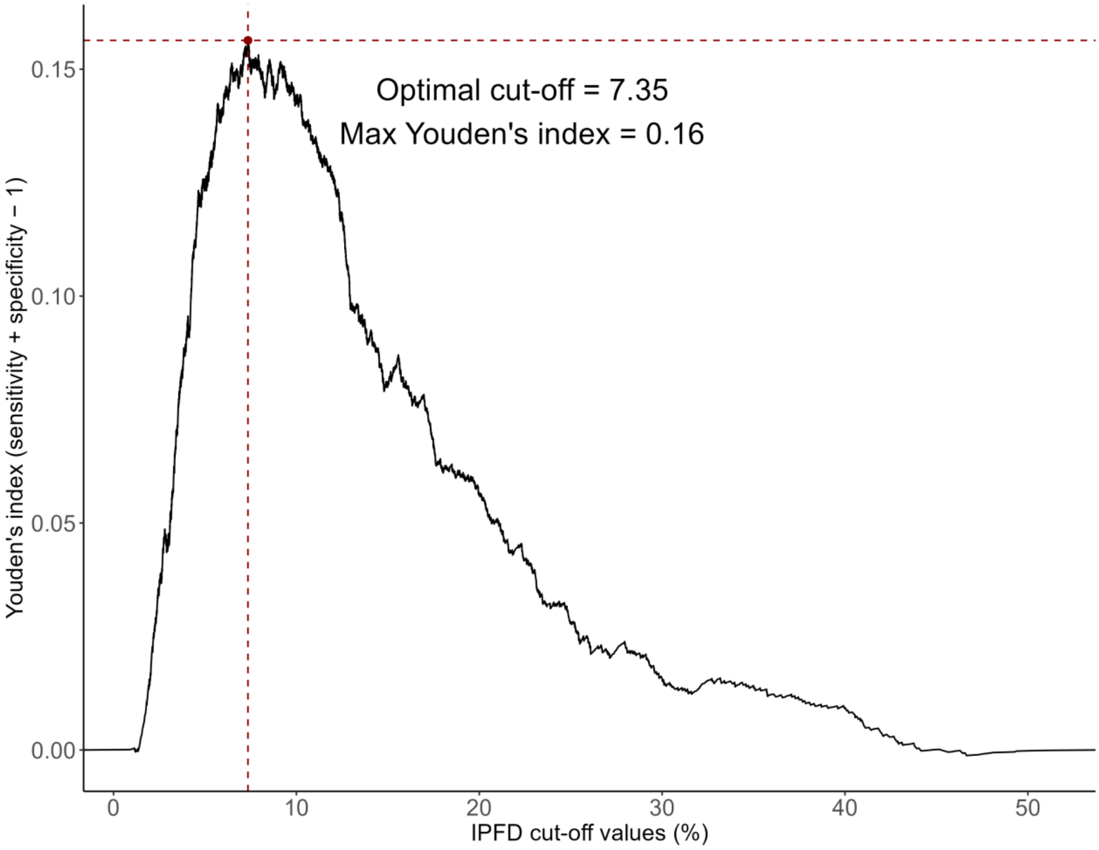

Figure S2. Determination of optimal intra-pancreatic fat feposition (IPFD) cut-off value using Youden's Index. The x-axis represents potential IPFD cut-off values (%), and the y-axis shows Youden's index (sensitivity + specificity – 1). The curve depicts how Youden's index changes with varying IPFD cut-offs. The optimal cut-off value, determined by maximizing Youden's index, is 7.35%, where the index reaches 0.16 (indicated by the red dashed lines).

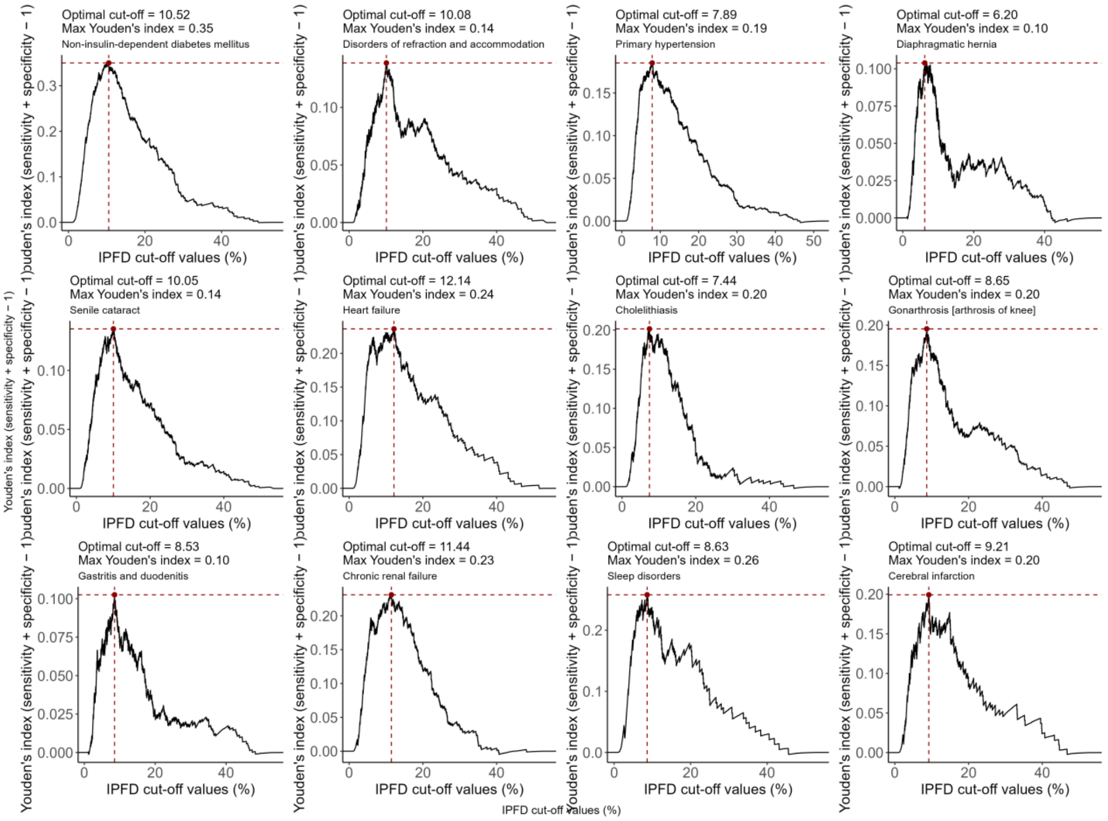

Figure S3. Determination of disease-specific optimal intra-pancreatic fat feposition (IPFD) cut-off value using Youden's Index. The x-axis represents potential IPFD cut-off values (%), and the y-axis shows Youden's index (sensitivity + specificity – 1). The curve depicts how Youden's index changes with varying IPFD cut-offs. The optimal cut-off value, determined by maximizing Youden's index, is 7.35%, where the index reaches 0.16 (indicated by the red dashed lines).
